# Healing Through Support: Beneficial and Detrimental Practices in Parental Grief—A Qualitative Study

**DOI:** 10.3390/bs15040535

**Published:** 2025-04-15

**Authors:** Lucía Pelacho-Ríos, Samuel Mayoral, María José Jorques-Infante, Gloria Bernabe-Valero

**Affiliations:** 1 Escuela de Doctorado, Universidad Católica de Valencia San Vicente Mártir, 46001 Valencia, Spain; lucia.pelacho@ucv.es; 2 Facultad de Psicología, Universidad Católica de Valencia San Vicente Mártir, 46100 Valencia, Spain

**Keywords:** parental grief, support practices, healthcare professionals, grief support groups, qualitative study, bereavement, post-traumatic stress, post traumatic growth, death, psychological intervention

## Abstract

This qualitative study explores the experiences of 24 parents who have experienced the death of a child, focusing on identifying practices that either facilitated or hindered their grieving process. In-depth interviews revealed key supportive practices, including emotional empathy, clear communication, and the presence of healthcare professionals during critical moments. Parents highlighted the significance of support groups, such as “Renacer”, in providing emotional connection, hope, and the opportunity for personal transformation. However, negative experiences emerged related to impersonal communication, lack of empathy, and delays in bureaucratic processes, which aggravated the pain and hindered emotional adjustment over time. The findings underscore the importance of personalized, empathetic care and the need for more efficient systems in supporting grieving parents. These insights can guide professionals in improving grief care, emphasizing respect for the emotional needs of parents and the creation of safe, supportive environments.

## 1. Introduction

*“Look, we even say that it’s not just mourning, because mourning falls short. Mourning is something that one eventually overcomes, and people go to therapy because they have experienced the death of a father, a mother, or even a husband (I say this because there are people in the group who have also faced the death of their partner). But the death of a child is on another level, it’s a different league. (It requires…) an extraordinary sensitivity because there is nothing left to say; everything has already been said, everything has been lived, and everything has been experienced. So, helping us as professionals is difficult.”*

Participant 16

The death of a child is among the most devastating experiences a parent can endure, profoundly shaking their sense of identity and purpose (Kim & Lee, 2021). Unlike other forms of bereavement, parental grief is marked by its overwhelming intensity, prolonged duration, and intricate complexity, often demanding specialized interventions and sustained support (Worden, 2018). Research has explored various approaches to assisting bereaved parents, including support groups, individual therapy, medical interventions, and broader community resources. Yet, a fundamental question remains: to what extent do these interventions truly fulfill their intended purpose?

The relevance of this question is underscored by several key factors. Parental grief has a lasting impact on mental and physical health (Stroebe et al., 2007). Second, contemporary societies often lack readiness and preparation to adequately address the unique challenges faced by bereaved parents, as child death has become a relatively rare occurrence in many developed countries (Cacciatore et al., 2008). Third, lack of sustained support can worsen grief and harm parental health (Snaman et al., 2016; Vig et al., 2021). Fourth, beyond emotional pain, grief also disrupts family dynamics, and while some bereaved parents seek specialized support, others find sufficient comfort within their existing support systems. Despite the severity of these issues, research on the effectiveness of available interventions remains limited, while reviews of culturally sensitive interventions highlight the need for standardized approaches and further research to enhance their efficacy (Aho et al., 2012; Vig et al., 2021; Aeschlimann et al., 2024).

Parents in this situation can rely on different resources, such as mutual support groups, individual psychological therapy, and direct care from medical professionals, to help them cope with their experience.

Support groups, which provide parents with the opportunity to share their experiences with other bereaved parents, have been shown to be beneficial. Specifically, these groups have been proven effective in fostering mutual understanding and normalizing grief experiences, as they help bereaved parents validate their parental identity and attain acknowledgement of their everyday life experience after the death (Clarke, 2015). Indeed, Kreicbergs et al. (2007) offered evidence that parents who engage in group-based discussions about their struggles during their child’s illness experienced a more meaningful grieving process.

Individual psychological therapy provides a more tailored approach, enabling the specific needs of each grieving parent to be addressed expressly and more effectively (Neimeyer & Harris, 2016). In this sense, theoretical models of grief have evolved over time, moving away from traditional frameworks that focus on “letting go” and toward contemporary approaches that emphasize maintaining a connection with the deceased child (Davies, 2004). Interventions grounded in these models include affirming parenthood, creating lasting memories, maintaining follow-up contact, and engaging in commemorative activities, all of which have a strong theoretical foundation and have been shown to benefit grieving parents (Kochen et al., 2020).

The role of medical professionals is also crucial in the initial stages of grief, especially in cases of perinatal or infant death (Gold et al., 2007). For instance, Van der Geest et al. (2014) observed that effective communication and continuous care during the child’s palliative phase can significantly reduce long-term grief levels in parents. In the same line, positive interactions with hospital staff and access to support resources during and immediately after the child’s death can positively influence parents’ subsequent grieving experience (Snaman et al., 2016).

Most existing studies focus on individual psychological responses and therapeutic interventions, yet there is growing recognition that a more holistic understanding of grief is necessary. Social, cultural, and political factors shape the grieving process in ways that extend beyond clinical settings, influencing the availability and effectiveness of support. For example, in non-Western contexts such as China, grief is deeply affected by cultural beliefs and social norms. The experience of Shidu parents—those who have lost their only child—is particularly complex due to the lasting effects of the one-child policy.

Shidu parents’ grief is profoundly shaped by cultural beliefs and social norms, such as the notion of fate and the stigma surrounding child loss, which can lead to somatic responses, feelings of inferiority, and an increased risk of depression and suicidal ideation (Ma et al., 2022; Zhao et al., 2021). Social support plays a crucial role in mitigating these effects, as greater support is associated with reduced depressive symptoms (Zhao et al., 2021). Similarly, within the Black community in the United States, communalism and community connections facilitate the grieving process, highlighting the significance of social ties in emotional recovery (McDuffie, 2022).

Given these variations, culturally sensitive grief interventions are crucial. Effective support must account for cultural norms, community structures, and the broader sociopolitical environment. A review of culturally competent grief treatments underscores the need for more research to develop standardized, adaptable approaches that meet diverse parental needs (Aeschlimann et al., 2024). Furthermore, beyond psychological and social support, the role of institutions such as hospitals and law enforcement is also significant. The presence of police officers and social workers in the early stages of grief, for instance, can either provide a sense of security or exacerbate distress, depending on how their involvement is perceived.

This study emphasizes the need for culturally competent grief support, advocating for interventions and policies that address the diverse needs of bereaved parents. By integrating cultural awareness, improving healthcare training, and informing policy changes, it seeks to develop more empathetic and effective support systems that help parents navigate their loss and rebuild their lives.

In summary, this study underscores the importance of culturally competent grief support, advocating for interventions and policies that address the diverse needs of bereaved parents. Effective support groups, individual therapy, and medical care play a crucial role in facilitating the grieving process and improving long-term outcomes. However, their effectiveness varies among parents (Stroebe et al., 2007), highlighting the need for research to identify both beneficial and counterproductive aspects. Through in-depth interviews with bereaved parents, this study aims to determine which practices are most effective, ultimately optimizing support systems, enhancing healthcare training, and informing policy changes to better assist grieving parents.

## 2. Materials and Methods

Given the innovative nature of this topic, this study adopted a qualitative approach, specifically employing deductive thematic analysis through in-depth interviews with parents to explore their grief experiences. This method is particularly effective for gathering comprehensive insights into areas with limited prior research. In the realm of psychology, qualitative methodologies are invaluable for delving into the intricacies of human behavior and subjective psychological processes, as well as the contexts in which they occur (O’Brien et al., 2014). Given the study’s goal to delve into personal attitudes, in-depth semi-structured interviews were selected as the most appropriate methodology. This approach enables researchers to explore specific topics outlined in a guide while affording interviewees the freedom to express their diverse thoughts and emotions. We also deemed qualitative methodology and interviews to be the most suitable tools due to the highly sensitive and personal topics here researched, such as the death of a child (Tong et al., 2007).

### 2.1. Participants

The sample included 24 parents who had experienced the death of a child, recruited through “Renacer” a support organization for grieving parents. Using purposive sampling, selection prioritized informative value over quantity (Malterud et al., 2016). Given their direct experience, participants had high “informative power”, making the sample suitable for generating meaningful conclusions. Participants’ most relevant characteristics are shown in Table 1.

Of the 24 participants, 19 were women and 5 were men, with an average age of 57.6 years (range: 37–76). Their children’s average age at death was 20.46 years (range: 4–44), and the time since death varied from 6 months to 13 years. Causes of death were diverse.

### 2.2. Research Team and Methods of Data Collection

A team of four clinical psychologists and researchers, specialized in existential and positive psychology, conducted this thematic analysis. Three held doctoral degrees, while the fourth was a doctoral student with extensive grief counseling experience. They managed interviews, transcriptions, and data analysis, with occasional support from postgraduate students trained in grief studies. Interviewers provided sensitive introductions and debriefings to support participants.

Inquiries during the interview addressed the experience of parental grief, as well as the factors that contributed to or hindered their bereavement process. To encourage open expression, interviewers established rapport before inquiring about bereavement. While following a structured script, they used additional probing questions when deemed necessary.

1. Did You Attend Therapy for This Reason?
  - Yes: Tell us about the aspects of therapy that you liked and found helpful. Share any aspects of therapy that you did not like and explain why.
  - No: Why did you not attend therapy? What would have helped you more?
2. How Has the Support Group Influenced Your Process?
  - How has the support group impacted your process?
  - What is it that helps you the most?
  - How do you feel when you identify with someone who has suffered the same death loss as you?
  - What aspects would you improve?
3. “What attitudes or behaviors from other professionals have you encountered that were particularly helpful or, conversely, that you would consider poor practices?”

### 2.3. Procedure

Prior to commencement, ethical approval was obtained from the Ethical Committee of the Catholic University of Valencia, under the project code UCV 2022-2023/024, ensuring compliance with the most rigorous ethical standards and regulatory requirements established for scientific research.

To initiate our research, we first reached out to Renacer association to present them with our proposal and arrange a pilot test for the interview protocol. This preliminary step was crucial for assessing the feasibility and relevance of the study by integrating the participants’ perspectives into the design of the data collection tools. After receiving approval from the association, we scheduled a meeting with two mothers who were members of the organization to conduct the pilot interview. This exercise enabled us to assess the clarity and comprehension of the questions, as well as to refine the language and duration in order to better align with the needs and characteristics of our target population.

Most interviews were conducted face-to-face, either at participants’ homes or in a dedicated, safe space at the university. For practical reasons, a few interviews were conducted online via video calls. Research team members and postgraduate students carried out the interviews and subsequently transcribed them. The study involved a Spanish-speaking population, and all interviews were initially conducted in Spanish. To ensure accuracy and preserve the richness of participants’ narratives, the interviews were transcribed verbatim in Spanish before being translated into English. The initial translation was performed by a bilingual researcher with expertise in qualitative research and grief-related terminology. A second bilingual researcher then reviewed the translation to identify any discrepancies and ensure that the original meaning remained faithful and natural in the context of qualitative analysis. Any differences were discussed and resolved collaboratively to maintain the validity of the study and minimize the risk of misinterpretation.

### 2.4. Data Analysis

The deductive thematic analysis in this study was informed by established theoretical frameworks that guided both the coding process and the interpretation of findings. Specifically, the analysis was structured around theoretical constructs relevant to grief and bereavement, ensuring that data interpretation was grounded in the existing psychological literature. The deductive approach allowed us to systematically identify and categorize themes based on prior knowledge, while also remaining open to emergent insights within the data (Ibrahim, 2012).

Our epistemological stance was rooted in pragmatism, emphasizing the integration of theory with real-world applicability (Kelly & Cordeiro, 2020). This perspective allowed us to draw on multiple conceptual models to frame the analysis, including, for example, Neimeyer’s approach to grief (Neimeyer & Harris, 2016), which highlights both the social and intrapsychic aspects of bereavement, focusing on resilience and addressing complications following the loss of a loved one. Additionally, we drew on Worden’s (2018) Grief Counseling and Grief Therapy: A Handbook for Mental Health Practitioners, which differentiates between grief counseling—aimed at guiding individuals through the natural tasks of mourning—and grief therapy, which addresses complications that disrupt the grieving process. This framework provided an interpretative lens for analyzing participant narratives, ensuring theoretical coherence in the coding categories and themes. Furthermore, it acknowledges the evolving nature of bereavement experiences and underscores the need for tailored interventions to support individuals in their grieving journey.

Furthermore, the deductive process was iterative and reflexive, allowing for the refinement of themes as new data or perspectives emerged (Fereday & Muir-Cochrane, 2006). While the analysis was initially guided by pre-existing theoretical constructs, we also remained attentive to unexpected patterns in the data, ensuring a balance between theoretical grounding and empirical openness. This methodological approach strengthened the study’s analytical depth by facilitating a structured yet adaptable interpretation of bereaved parents’ experiences.

Prior to analyzing the interviews, the research team convened several meetings to align themes, codes, and resolve uncertainties, following Boyatzis’ (1998) recommendations: (1) establishing themes through reading and theory, (2) checking the compatibility of themes with the data, and (3) determining coder reliability. This collaborative approach aimed to minimize subjectivity and enhance objectivity in the analysis process, as recommended by Franklin and Ballan (2001).

After a comprehensive analytical approach, a cross-verification system was implemented to ensure the reliability and consistency of interview coding. The process began with an in-depth, iterative reading of the interview transcripts to achieve a comprehensive familiarization with the data. This was followed by an open coding phase, during which we systematically identified and labeled salient patterns and conceptually significant elements relevant to the research objectives.

To enhance the rigor and consistency of the analytical process, the research team engaged in weekly meetings to review individual codings, resolve discrepancies, and refine the thematic structure. Through this iterative process of discussion, comparison, and refinement, we reached a consensus on the final set of themes and subthemes, ensuring that they provided a coherent and comprehensive representation of the participants’ lived experiences. Moreover, the analysis incorporated an examination of contradictions and divergent cases. These instances were critically reviewed and discussed within the research team to ensure that the thematic framework accounted for the complexity and variability of participants’ perspectives.

Through an iterative and collaborative approach, the study’s thematic structure was progressively refined. The final result was the development of a master compendium that captured the primary themes and their ramifications, supported by verbatim quotes extracted directly from interview transcriptions. This document served as a fundamental basis for interpreting and presenting the study’s findings.

Throughout the analysis, we engaged iteratively with the entire dataset, continuously identifying themes and subthemes that deepened our understanding of bereaved parents’ experiences. While certain patterns recurred across interviews, new nuances and perspectives continued to emerge, reinforcing the decision to analyze the full dataset. This approach aligns with contemporary discussions on the fluid and evolving nature of thematic emergence in qualitative research.

The execution and reporting of this study conform to the Consolidated Criteria for Reporting Qualitative Research (COREQ) (Tong et al., 2007).

Finally, to ensure the rigor and trustworthiness of our analysis, we implemented several quality assurance procedures. Reflexivity was maintained throughout the research process by engaging in continuous self-examination and critical reflection on our positionality, potential biases, and their influence on data interpretation. Researcher triangulation was employed through collaborative analysis, where all the researchers independently coded and reviewed the data before engaging in discussions to reconcile discrepancies and refine themes. As mentioned, dependability was enhanced by maintaining consistency in the coding process through iterative discussions and cross-validation within the research team. Credibility was reinforced by ensuring that themes were supported by rich, illustrative excerpts from participant narratives. Confirmability was achieved by systematically documenting the analytical process, including coding frameworks, thematic development, and decision-making rationales, ensuring that findings were traceable and could be reviewed by external researchers. Additionally, the use of direct quotations from participants helped maintain transparency and reduce the risk of researcher bias in the interpretation of the data. These strategies collectively strengthened the transparency, coherence, and validity of our qualitative analysis.

## 3. Results

**Figure 1:**
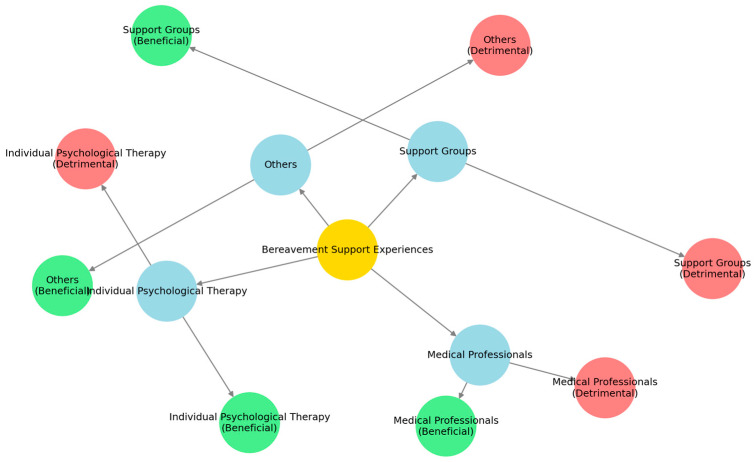
Visual map of themes and subthemes.

Our analysis identified both major themes and subthemes. Specifically, four overarching themes emerged: (1) support groups, (2) individual psychological therapy, (3) medical professionals, and (4) others. Within each of these themes, we identified both beneficial and detrimental subthemes, reflecting the nuanced and multifaceted nature of participants’ experiences (Figure 1).

### 3.1. Support Groups

#### 3.1.1. Beneficial Practices

Support groups were revealed as transformative spaces, offering not only comfort but also tools and a propitious environment for parents to process their pain and, eventually, transform it into love and personal growth. First and foremost, participants generally highlighted the acquisition of both human and spiritual tools that helped them navigate their grief. The weekly reading of inspiring texts followed by reflection was mentioned as a particularly useful practice.

“Renacer has provided me with both human and spiritual tools to accompany me on this journey.”

“I find it very positive that we always start by reading something from a book, and it’s also really thought-provoking that they post a text on WhatsApp every week—I think it’s wonderful.”

“They send us the texts every Monday, it works great for me. Because I read them, I write them down, I copy them into a notebook, almost like I’m studying them, and with that, I get through the week.”

Moreover, a crucial aspect was the feeling of being understood and therefore less lonely. Parents expressed profound relief upon finding others who lived through similar experiences, alleviating their sense of isolation. Indeed, mirroring themselves in the stories of other parents provided significant emotional validation.

“I love being with them, I feel very understood. It’s like my new life.”

“Renacer was really helpful because it stopped me from feeling alone”

“Well, knowing that everyone there has experienced the death of a child makes you feel like you’re not the only alien in the world or the most unfortunate.”

“You feel alone, thinking, ‘Wow, I’m the only one’. ‘I’m the only one who has to live through this’. Then, you realize that so many people are going through the same thing, and that helps a lot, it really helps a lot.”

Another aspect frequently mentioned as beneficial was seeing parents further ahead in their grieving process, as this inspired hope in newcomers. Observing that others had managed to rebuild their lives and find happiness again offered an encouraging perspective on their own future.

“Well, it gives hope. Seeing those who are further along than I am, who have spent more time in their process, seeing that they’re moving forward, some of them are very strong, with a lot of strength, a lot of power, a lot of optimism. I want to be like them.”

“Being with parents who had suffered the same as you, and seeing them doing well, it was good for me. Because I thought, ‘Wow, I can get to be that well.’”

Support groups also fostered an environment of mutual aid, where the act of supporting others became a source of strength and personal growth for grieving parents. This reciprocal aspect of support was highlighted as particularly rewarding in the following testimonies.

“You don’t do it to help yourself, but you realize that by helping, you’re the one who benefits the most.”

“And it’s about sharing, mutual help, because we help each other. I help you, but at the same time, by stepping out of myself, I’m helping myself.”

“That’s the greatest thing there is, it’s an unconditional love that you give and see that another person receives it. Wow! How beautiful it is to be able to help!”

Finally, many parents described how support groups helped them “rebirth”, making it easier to build a new life and reframe their death loss. This process involved learning to smile again, freeing themselves from feelings of guilt, and developing a more positive perspective on life and death.

“Renacer has been very important in my life. It made me smile. It made me see that my daughter is not my executioner, and it made me love life more. It took away the feeling of guilt. Since I’ve been going to it, I’ve realized that I can laugh peacefully.”

“Renacer is where I found the energy, the strength, the ability to see life differently, to see death differently. It’s like starting over. It’s a light that illuminates me, and I move forward, finding affection, love, understanding, and unity. Just the fact of going there, seeing people, hugging, and feeling the love—it’s all for our children.”

“So, in Renacer, I see people who are starting to enjoy things again and not punishing themselves for enjoying certain things. it restores the right to enjoy life.”

“What we want is to be well, process that pain and transform it into love.”

#### 3.1.2. Detrimental Practices

The analysis of the interviews revealed areas for improvement in support groups, according to the perceptions of participating parents. In particular, two main themes consistently emerged: a sense of stagnation and repetition and the need for more individualized support. Indeed, several parents expressed concern about the repetitive nature of the meetings, especially as the group grew in size. The growth of the group was also identified as a factor potentially affecting the dynamics of the meetings. Relatedly, parents further along in the grieving process expressed the feeling of being unable to progress due to the constant need to accommodate new members.

“We repeat ourselves a bit. We all speak, and there are so many of us, so when we discuss a topic, well, at some point, you feel like… I can’t contribute anymore because everyone has already spoken and practically said the same thing.”

“We’ve doubled in the last two years. Yes, now there are too many of us. It starts to overwhelm me a bit.”

“You can’t move forward, because you have to match the timing of those new parents.”

Secondly, the suggestion of implementing a more personalized support system also emerged regularly.

“They could ask you: hey, would you like someone to be a little more attentive towards you?”

“I think there could be groups of, I don’t know, three, four, five, six, seven people who decide, hey, we can be mentors or companions.”

Ultimately, these observations highlight the importance of continuously adapting the structure and dynamics of support groups to meet the changing needs of their members, balancing support for newcomers with the ongoing progress of more experienced participants.

### 3.2. Individual Psychological Therapy

#### 3.2.1. Beneficial Practices

In the area of individual psychological therapy, a prominent theme emerged too: the parents’ appreciation of active listening skills. Numerous testimonies emphasized how the mere act of feeling truly heard and having a safe space to express themselves in a professional care context provided invaluable support in their grieving process. This indicates that therapists’ ability to create a safe environment of trust and emotional containment represents a fundamental therapeutic element, highlighting the importance of the quality of the therapeutic relationship in supporting parental grief.

“But the good thing was that she listened to me, so that moment was good for me. It’s important to talk and be heard, it’s not about being asked questions. I now know that the answer is within all of us, so there has to be a professional who helps you talk, but who doesn’t question you…”

“Making you feel like you’re really not in front of a professional, but rather in front of someone who is listening to you and isn’t going to charge for it, you know? I don’t know, maybe it’s just my perception… but it’s about feeling comfortable with that person and knowing they’re truly listening… that’s really important.”

“I spoke with the director of the psychology service, she was a lovely woman, she dedicated almost an hour of her Sunday morning to me. I mean, these things are so important.”

The empathy and emotional validation provided by mental health professionals similarly emerged as crucial aspects in the experience of parents. Grieving parents emphasized how professionals’ ability to tune into their pain, acknowledge the legitimacy of their emotions, and offer genuine understanding substantially contributed to their healing process. This therapeutic approach, characterized by a deep understanding and acceptance of the mourner’s experience, appears to have played a fundamental role in creating an environment conducive to grief processing and emotional recovery.

“The calm with which she listened, validating everything, or whatever it was, validating any type of feeling.”

Throughout the course of the interviews, another significant theme emerged in the narratives of grieving parents: the positive appreciation of practical interventions offered by mental health professionals. In this sense, participants highlighted the importance of receiving concrete guidelines and specific tools as fundamental elements in their coping and recovery process. These strategies, tailored to the individual needs of each mourner, were perceived as tangible anchors amid the emotional turbulence of grief. The provision of these practical resources not only facilitated the management of challenging daily situations but also empowered parents, providing them with an increased sense of control and the opportunity to take action in their journey through grief. This finding stresses the relevance of a therapeutic approach that combines emotional support with pragmatic, action-oriented interventions.

“I liked it when they told me I had to go home before my daughter passed away. The psychologist told me: go home before your daughter dies because it will be harder to go after. So, it helped me because later, this made it easier for me.”

“What I liked most therapeutically about the psychologist is that she was able to combine different tools from various approaches.”

“She provided audios and exercises, which have been very helpful in my therapy.”

“When you’re feeling so bad, so bad, and you see someone dedicating their time to you and giving you a roadmap on how to deal with something you don’t know how to deal with…”

One last notable finding emerged regarding the use of pharmacological interventions during the grieving process. In this vein, several participants explicitly and positively highlighted the beneficial role that prescribed medications played during the most intense periods of grief, providing temporary relief from the overwhelming emotional and cognitive burden that accompanies the death of a child. The medication was perceived as a complementary tool that facilitated navigating the most turbulent moments of sorrow, offering the necessary stability to engage in other forms of therapy and begin the challenging process of emotional reconstruction. This finding underscores the importance of considering a comprehensive approach to the treatment of parental grief, where pharmacological interventions, when appropriate and carefully managed, can play a significant role in alleviating suffering and promoting resilience.

“So I went to the psychiatrist, and she prescribed me antidepressants, which I’m taking, of course. And then, Orfidal to sleep, which I take because if not, I would wake up and be horrified thinking that my child is no longer here.”

“When my son died, I went to the psychiatrist, and she gave me some pills which I took for at least a year because I was overwhelmed by how badly I was feeling.”

“The pills give you strength, I mean, I feel like the pills put me a few steps above where I was emotionally, they gave me a strength that I wouldn’t have had on my own.”

#### 3.2.2. Detrimental Practices

In spite of the positive elements described above, the analysis of participants’ responses also revealed an inconsistent landscape regarding the interventions of mental health professionals. Indeed, while many practices were positively evaluated, testimonies also highlighted practices perceived as misguided or even counterproductive. Some participants described therapeutic encounters that, rather than providing relief, exacerbated their emotional distress during this period of extreme vulnerability.

In the analysis of the grieving parents’ experiences, a significant theme emerged relating to the prescription of psychiatric medication. Indeed, several participants expressed disagreement with the pharmacological approach suggested by some mental health professionals, perceiving this intervention as unsuitable for their grieving process. The following comments reflect a dissonance between the parents’ perceptions of their needs and the treatment offered, suggesting a misalignment between professionals’ approach and mourners’ expectations.

“I went to a psychiatrist, and he wanted to give me pills. And I thought, I don’t need this.”

“The psychiatrist asked me three questions and then said, ‘You’re going to take this…’ And I was like, ‘What are you telling me? With this little pill, is my pain going to go away?’ And he said, ‘No… but it will help.’ And I said, ‘No, no, I don’t want it to help, I want to understand what I’m going through, and I measure myself every day to see if I’m doing better or worse. If you give me a pill, I won’t know if it’s me or the pill.’ So, I never went back.”

Our analysis also revealed other significant experiences of grieving parents regarding certain practices of mental health professionals, which were perceived as inadequate or counterproductive and described by participants as unprofessional or insensitive.

“I went to a psychologist, but he started crying when I told him what had happened to me.”

“But I really don’t like it when, before we even start, they bring out the tissues. They should be hidden because if you assume the person is going to cry, I don’t think it’s very delicate, you know? Of course, you’re going to need tissues, but they should be hidden, and it should be more like, ‘Oh, look, here I have them,’ rather than saying, ‘Hey, you’re going to cry, you’re here to cry.’ It’s not sensitive.”

“But the psychologist never got to what needed to be addressed.”

“I told him that I had discovered Renacer and that I thought it would be better for me, and he looked at me and said ‘Well, yes, that won’t help you, don’t tell me nonsense…”

In addition to the themes above, several participants mentioned the imposition of unwanted therapeutic techniques or negative experiences with specific therapies.

“I’m doing EMDR, which is a very tough therapy, and right now, I still don’t see the benefit. It feels like falling into hell.”

The analysis of the interviews also revealed a recurring and concerning pattern: the perception among grieving parents of mental health professionals’ inability to truly understand the depth and uniqueness of their pain. This lack of understanding emerges as a significantly detrimental factor in the therapeutic process. In the same line, multiple testimonies highlight the frustration experienced when interventions seemed to minimize or misinterpret the magnitude of their death loss. Relatedly, the feeling of receiving generic and inadequate treatment was common among participants. Indeed, the perceived ineffectiveness of some interventions, which were mostly based on a theoretical description of grief without offering practical tools or meaningful emotional support, was considered insufficient and disconnected from the real needs of the parents.

“Don’t you understand what I’m talking about? I mean, the death of a child is something else… it’s something apart, it’s not categorized. It doesn’t follow those guidelines.”

“No, I think that if you haven’t gone through this situation, you don’t know how hard it is. The advice she gave, I suppose she gives it to everyone, I don’t know.”

Particularly problematic was the application of standard therapeutic concepts without the necessary adaptation to the context of parental grief. This comment reflects how certain interventions, far from providing comfort, may be perceived as offensive or completely disconnected from the mourner’s reality.

“I don’t know about this idea of acceptance, I wanted to kill them when I heard ‘acceptance,’ what are you talking about? Or ‘be a better person’, what are you saying? I was already a good person. I didn’t need my child’s death to be a better person.”

“It didn’t help us at all, you know? They just told us a bit about what grief is and the stages we had to overcome.”

### 3.3. Medical Professionals

#### 3.3.1. Beneficial Practices

The analysis of the interviews exposed several practices by hospital staff that grieving parents perceived as appropriate and beneficial during the critical moment of losing their children. These practices were grouped into three main categories.

First, allowing parents’ presence in the hospital, making it possible for parents to say goodbye to their children. Parents deeply appreciated the permission to stay close to their children in the final moments, especially when hospital regulations would typically restrict such access.

“They allowed us to enter the ICU… to be with her, and in the end, that is something we are really grateful for because we were able to say goodbye to her.”

Second, the way in which the event of the child’s passing was communicated was crucial for parents. In particular, gestures of compassion and clarity facilitated parents processing of such difficult news during an extremely vulnerable moment. Parents especially appreciated clear and straightforward communication, comforting physical touch, and the empathetic attitude of staff, as evidenced by the following quotes.

“When they told me, they did so with a lot of affection. They held my hands, they hugged me…”

“Even one of the nurses hugged me.”

The empathetic attitude of the staff:

“The doctor who told us was very kind.”

Third and last, parents positively valued the offering of psychological support and additional services, as these offerings we perceived as demonstration of holistic concern for parents’ well-being beyond immediate medical care.

“He was attended to by the doctors, and they were wonderful, with great sensitivity…”

“The professionals were amazing, I even called them to thank them and tell them how well they had done.”

“The ambulance arrived very quickly.”

#### 3.3.2. Detrimental Practices

The analysis of the interviews also revealed several practices by medical professionals that parents perceived as inadequate or harmful during the critical moment of losing their children. These negative experiences generally related to poor communication abilities regarding the death of their children, the treatment received beyond specific communications, and delays in services—factors that parents described as compounding their pain and confusion at this difficult time.

“Everything was either too abrupt or too distant… very objective.”

“I don’t remember such an unfriendly nurse in my entire life, and I don’t think I will ever meet any nurse as unfriendly as that one. It was very unpleasant.”

“I would have needed a more caring approach, not so technical from the doctor, a bit more warmth.”

“The feeling was that the doctor took the bitter pill of having to communicate the bad news to us himself.”

### 3.4. Others

The analysis of the interviews revealed that, in addition to hospital staff and mental health professionals, other professionals played a crucial role in supporting parents during the time of their death loss. Two groups of professionals were particularly highlighted for their effective practices: police officers and social workers.

#### 3.4.1. Beneficial Practices

Parents expressed deep gratitude towards police actions, highlighting two main aspects: (1) constant presence and (2) active accompaniment. This continuous presence and proactive accompaniment provided the parents with a sense of security and support during times of extreme vulnerability.

“The police did a phenomenal job too because they were there from the beginning; they arrived right away.”

“They accompanied the ambulance all the way.”

As anticipated earlier, the intervention of social workers was also perceived as highly beneficial, particularly in terms of providing timely and crucial information as well as practical guidance. These timely and practical interventions by the police and social workers proved invaluable for the parents, providing not only emotional support but also practical assistance in moments of extreme need.

“The social worker came and informed me, saying, ‘You know your child is a minor, and you have the right to request leave for childcare services.’ So, it was a huge help when someone came and explained it to me.”

#### 3.4.2. Detrimental Practices

However, the analysis revealed a particularly painful aspect in the management of the death of a child: delays in bureaucracy and communication of the death. A notable testimony illustrates this issue.

“The Spanish embassy didn’t communicate it until 7:30 in the morning, but she passed away at 2:30 in the morning. I think they should have paid a little more attention, as my family was waiting.”

The delay in official notifications not only prolongs the anguish of the parents but also potentially delays the onset of the grieving process and necessary arrangements. This aspect highlights the need for more efficient and empathetic protocols in the communication of deaths, particularly in international contexts.

### 3.5. The Influence of External Factors on Parental Grief

In interpreting the results of this study, it is important to consider how various external factors might affect the grieving experiences of parents who have lost a child. While emotional and psychological impacts of parental grief are well documented, the ways in which public health policies, social and cultural contexts, and access to mental health services might influence these experiences are equally critical, particularly when evaluating the professional practices parents encounter. These factors play an essential role in shaping how parents receive, experience, and process support, both positive and negative, from professionals during their grieving process.

#### 3.5.1. Public Health Policies and Professional Support

Public health policies in Spain play a critical role in shaping the availability and quality of bereavement support. However, regional disparities significantly affect both access to services and professional practices, leading to inconsistencies in the care provided to grieving parents. The effectiveness of bereavement care largely depends on the presence of structured grief support programs and the extent to which healthcare professionals are adequately trained to address grief-related needs. When such programs are lacking, or when professionals are not sufficiently equipped, parents may experience inadequate or even harmful care, exacerbating their distress.

The qualitative data from participant interviews highlight the importance of resources and systemic support in navigating bereavement. Several participants acknowledged their privileged position within a high-income country, recognizing that their access to grief-related resources was largely secured. One participant expressed the following:

“We are privileged. We live in the first world, let’s not forget that. We experience grief in a comfortable way, from a position of privilege. Our material conditions are secured, I could allow myself to take leave.”

Another participant reinforced this sentiment, emphasizing the benefit of flexible leave policies:

“Fortunately, I was able to take leave until I felt strong enough to return to work.”

However, while some parents were able to access psychological support, this access often depended on financial resources, which are not always an option. One participant described their experience:

“Let’s be honest, in this regard, we are privileged because whenever we needed a psychologist, we had one.”

This acknowledgment underscores the disparity between those who can afford private care and those who must rely on public services, which may be limited or inconsistent. Another participant explicitly linked bereavement support to financial means:

“Material resources are important, you can afford a psychologist.”

Despite the availability of support in some cases, the bureaucratic barriers within the healthcare and administrative systems often added an additional layer of distress, directly impacting the professional support received by bereaved parents. One parent described an encounter with an unempathetic administrator, illustrating how procedural inefficiencies and a lack of sensitivity from professionals can exacerbate grief:

“The first reaction from the clerk was unpleasant. It was like, just fix this, don’t add more problems, please. Don’t burden us with bureaucracy and complications. It’s already painful enough to making it worse.”

This interaction highlights how the effectiveness of professional support is not only determined by access to psychological or medical care but also by the responsiveness and compassion of all professionals involved in the bereavement process. When frontline professionals, including administrative staff, lack training in grief-sensitive communication, they may unintentionally contribute to parents’ distress rather than alleviating it.

These testimonies illustrate that while some bereaved parents benefit from structured support, others encounter obstacles that compound their grief. The role of healthcare professionals is crucial in mitigating these challenges. Ensuring that professionals receive adequate training in grief-sensitive care and that bereavement policies are standardized across regions could enhance the quality of support available to grieving parents. Additionally, reducing bureaucratic burdens and fostering a culture of empathy within public services could significantly improve the experiences of those navigating parental loss.

Ultimately, the findings underscore the need for a more equitable and accessible bereavement care system in Spain—one that considers both the structural disparities and the individual experiences of grief.

#### 3.5.2. Social and Cultural Context in Professional Practices

The social and cultural context in Spain plays a fundamental role in shaping healthcare practices and parental grief, with Catholicism being a particularly influential factor. Deeply embedded religious beliefs surrounding death often shape societal attitudes toward mourning and professional support. These cultural frameworks can influence how professionals provide care to grieving parents, sometimes reinforcing traditional norms that may not always align with individual experiences. As one participant expressed:

“I wish someone had properly guided me on the right path to follow when… when my son was in his final stage. I felt that a child should be accompanied differently. I don’t know what they should have told me, but I needed more from them.”

Another parent emphasized the absence of professional support:

“Well, yes, excuse me. I missed a call from his doctor to offer condolences. I just don’t understand how a professional who had been treating a young person for two and a half years wouldn’t reach out after his death.”

However, these cultural frameworks can also provide essential emotional and social support, offering grieving parents a sense of structure, meaning, and community during an incredibly difficult time. Mental health professionals play a crucial role in navigating this balance. They must acknowledge the potential benefits of cultural traditions while ensuring that their interventions remain sensitive to the unique needs of each individual.

“They told me that my daughter is fine, that she is in heaven, and that helped me feel better.”

In regions where family and community support networks are particularly strong, healthcare professionals are more likely to recognize and encourage their involvement. These networks can significantly enhance the grieving process and improve the quality of care provided to parents.

“And then, other resources that are also very relevant are family resources. My family was incredibly supportive. Both families came together, and that was extremely important.”

“No, at first, I had none—no capacity at all. I had no personal capacity. You are not yourself anymore. You cease to exist. You have to surround yourself with people… The people around you are the ones who pull you through. You have no resources, only pain.”

One participant highlighted the importance of community support:

“What helped me tremendously was being involved in the Asprona Board because of my disabled son. I felt so, so, so supported and loved there. The group strengthens you, fills you, and sharing with others has helped me a lot.”

In conclusion, understanding external factors such as public health policies, social and cultural contexts, and access to mental health services provides a more comprehensive view of the professional practices parents encounter during their grief process. These factors significantly influence the quality of care they receive, either facilitating or hindering effective grief support. By considering these broader influences, this study highlights how professional responses to parental grief in Spain are shaped by both the healthcare system and the wider social and cultural environment.

## 4. Discussion

The goal of the present research was to gain insight into bereaved parents’ perceptions of beneficial as well as detrimental support practices, with a focus on support groups, individual psychological therapy, and medical professionals. This is an important endeavor as this knowledge is essential for understanding how healthcare professionals, those managing support groups, and other practitioners can enhance the quality of the support offered to families during this profoundly painful process. Based on the testimonies gathered among 24 bereaved parents, the current research identified a number of practices that those parents perceived as helpful or, contrarily, contributing to additional harm. In the following sections, we summarize and discuss such practices.

### 4.1. Beneficial Practices

One of the most valued practices was the empathy and emotional support provided by professionals and support groups. This finding aligns with Job’s et al. (2022) recommendation, who recently emphasized the importance of addressing the social and spiritual dimensions of parental grief. The emotional connection and acknowledgment of shared pain in support groups, such as Renacer, were also highlighted as key elements helping parents feel understood and less alone in their suffering, a finding also consistent with prior research (Berrozpe et al., 2024).

Support groups, such as “Renacer”, emerged as transformative spaces offering human and spiritual tools to navigate grief. Notably, the most valued aspects of these groups included (1) acquiring tools to process pain, such as reading and reflecting on inspiring texts; (2) the feeling of being understood and less alone by sharing with others who have lived through similar experiences; (3) the inspiration and hope derived from seeing parents further ahead in their grieving process; (4) the opportunity to help others, which became a source of personal strength; and (5) the process of “rebirth”, which involves building a new life and reframing the death of the child. These findings are in line with a recent systematic review examining beneficial practices for accompanying parental grief, which similarly highlighted the advantages of meaning-centered therapy and emphasized the importance of helping parents find meaning in their death loss and rebuild their lives), which closely aligns with the transformative experiences reported by parents in support groups like “Renacer” (Pelacho-Rios & Bernabe-Valero, 2023).

Experiences with mental health professionals in the context of individual psychological therapy revealed several crucial aspects. First, the importance of active listening and creating a safe space for emotional expression was emphasized. Additionally, as Kruse et al. (2024) point out, empathy and emotional validation were identified as fundamental elements in the therapeutic process, as these are often absent (or scarce at best) in parents’ social circles. Furthermore, participants also positively valued practical interventions and specific tools that helped them cope with challenging situations in their everyday lives. Lastly, in line with the previous literature, the beneficial role of medication was observed in some cases, providing crucial support during the most intense periods of grief (Lacasse & Cacciatore, 2014). Notwithstanding, it is important to note that several parents also expressed negative experiences with medications prescribed by mental health professionals, an issue that we return to below when discussing detrimental practices.

Medical professionals also played a crucial role in the parents’ experience. Here, three aspects were highlighted as particularly beneficial: (1) allowing parents to be physically present and therefore able to say goodbye, even when hospital regulations would normally restrict it; (2) compassionate and clear communication of the death, which included comforting physical contact and an empathetic attitude; and (3) the offering of psychological support and additional services, demonstrating a comprehensive concern for the parents’ experience and well-being. These practices attest to the importance of empathy and flexibility in hospital settings during such painful moments, something similarly observed in prior studies (Donovan et al., 2015). Hospital-based grief services reduce isolation, improve coping ability, and promote personal growth in parents who experience more complex grief after the death of a child. Indeed, participants’ testimonies reflect that an empathetic approach to communication can facilitate the grieving process, mitigating the impact of pain and allowing parents to process their children’s death more effectively. These results are consistent with those of Turner et al. (2022), who found effective communication with healthcare professionals after the death of a child to influence parents’ grief experience significantly.

Finally, the actions of the police and social workers emerged as significant factors in the early stages of grief. The constant presence and active company of police officers provided parents with a sense of security during moments of extreme vulnerability. Social workers, on the other hand, offered crucial information and practical guidance, demonstrating the importance of comprehensive support addressing both emotional as well as practical needs.

### 4.2. Detrimental Practices

On the other hand, negative experiences highlight practices that parents perceived as harmful. Perhaps the most critical aspect was the lack of empathy and coldness in communicating children’s death. Many parents reported that interactions with some professionals were perceived as impersonal and technical, which intensified their pain. The feeling of being treated with distance and without the necessary emotional care can exacerbate the already devastating suffering of losing a child. This is evident in the study by Butler et al. (2017), in which grieving parents identified behaviors such as minimizing their concerns, the improper management of hope, and a lack of professionalism as increasing emotional suffering and the sense of isolation among parents. Relatedly, the perception of superficial evaluation by some professionals, such as psychiatrists, was observed, which was reported to generate frustration and distrust in grieving parents.

Parents’ testimonies also reflected concerns about the potential interference of medication in their ability to assess their own emotional progress. Indeed, several parents felt that the use of medication could cloud their experience of grief, preventing them from recognizing their progress or setbacks. It is essential that the support provided is perceived as beneficial and respectful of each person’s individual process, as many participants expressed a deep desire to consciously experience and process their pain, seeking a space where their emotions are validated and understood—rather than simply put away by medication. This stresses the importance of individualized and sensitive approaches to parental grief treatment, where proposed interventions must carefully align with the needs and preferences expressed by the bereaved, a view previously expressed by Klasen et al. (2017).

Bureaucratic delays were also highlighted as a damaging factor. Delays in the official communication of the death seemed to prolong parents’ experience of distress. This bureaucratic inefficiency not only increased immediate suffering but could also negatively impact the initiation of the grieving process.

Regarding psychologists, our research suggests that inadequate psychological practices in parental grief include (1) lack of continuous support, (2) poor communication, (3) absence of systematic follow-up, and (4) variability in grief services, which can lead to negative outcomes in the parents’ mental and physical health. Additionally, a lack of proper training among some psychologists in managing parental grief was identified, which prior research has identified as an antecedent of inadequate support during the grieving process of bereaved parents (Vig et al., 2021), often prompting parents to discontinue treatment or seek help elsewhere. This is important because inadequate practices from professional psychologists can often lead to deterioration of the psychological and physical well-being of grieving parents, something that has also been observed by various authors (Guldin et al., 2013; T. October et al., 2018). Crucially, the negative impact of inadequate practices can extend in the long term, affecting parents’ ability to recover and adapt after the death of a child, thereby increasing the likelihood of experiencing pathological grief, as previously noted by T. W. October et al. (2014).

### 4.3. Implications for Practice

This qualitative research on parental grief provides valuable insights into improving support for bereaved parents, offering several key practical implications for health professionals. A primary recommendation is the need for ongoing, empathy-focused training for both mental health and medical professionals. This training should go beyond theoretical understanding, equipping professionals to recognize and respond to the unique and profound impact of child death loss. By validating diverse grief experiences and coping strategies, professionals can develop personalized interventions that respect individual differences. Additionally, understanding the long-term mental health outcomes of parental grief is essential, as unresolved grief can lead to chronic conditions such as depression, anxiety, or PTSD. Tailored therapeutic interventions, such as grief counseling or support groups, could help mitigate these outcomes, fostering better emotional recovery for parents. By enhancing professional practices with these insights, the quality of care for grieving parents can be significantly improved.

Cultural differences play a significant role in how bereaved parents experience and cope with grief, and it is important for professionals to understand these variations when offering support. For example, in some cultures, emotional expression may be seen as a sign of weakness, while in others, public displays of grief are an important part of mourning. In certain cultures, spiritual beliefs may offer comfort and structure during the grieving process, whereas in others, grief may be handled more privately, and parents may feel a sense of shame in seeking external help. Therefore, understanding the cultural context in which grief occurs is crucial for health professionals to offer appropriate, sensitive care that aligns with the values and norms of bereaved parents.

To ensure culturally competent care, training programs for health professionals should include a focus on cultural awareness, helping them understand how grief is influenced by factors such as religion, societal expectations, and family dynamics. This awareness allows professionals to tailor their support practices to the needs of parents from diverse cultural backgrounds, ensuring that the support provided is meaningful and respectful. Culturally informed practices may include being mindful of communication styles, respecting traditional mourning rituals, and recognizing the role of extended family or community networks in the grieving process.

The study also emphasizes the importance of long-term support systems. Parental grief often extends well beyond the immediate aftermath of death, requiring ongoing emotional and practical assistance. Health professionals should be trained to provide continuous care, ensuring that grieving parents are not left without support as time passes. Additionally, creating safe spaces for parents to express emotions and share experiences, as well as adopting more sensitive communication protocols when delivering difficult news, is vital in mitigating the emotional burden during the grieving process.

In terms of practical guidance, specialized training for healthcare professionals is crucial in recognizing the signs of prolonged or complicated grief and responding appropriately. For parents who exhibit severe symptoms, psychiatrists should discuss medication options, providing clear explanations to empower informed decision-making about treatment.

Moreover, public health policies at the national and regional levels must play a role in influencing social support systems. Policymakers should prioritize the integration of grief support services within healthcare frameworks and ensure that sufficient resources are allocated to these services. By implementing policies that support comprehensive grief care, we can ensure that grieving parents have access to both emotional and practical assistance, improving their overall well-being.

The study also underscores the significance of multidisciplinary support, particularly the actions of police officers and social workers in the early stages of grief. The presence and active involvement of police officers during critical moments provided parents with a sense of security, while social workers offered essential information and practical guidance. Their role highlights the importance of comprehensive support that addresses both emotional and practical needs, particularly in the initial, most vulnerable stages of grief.

Finally, ongoing research is vital to refining care practices and improving the support provided to grieving parents. By continuously exploring the unique needs of bereaved individuals and integrating these insights into practice, we can enhance the quality of care, mitigate complications in grieving, and foster healthier recovery paths, ultimately offering compassionate and individualized support for those facing profound death loss.

### 4.4. Limitations and Future Research Directions

The present research has limitations that should be acknowledged and can serve as avenues for future research. First, our sample was comprised exclusively of parents attending Renacer group, which may not reflect the wider diversity of grief experiences and opinions of other parents. As such, although the testimonies gathered provide valuable insights into the practices and resources that benefited these parents, there may be a certain degree of selection bias in our sample—participants, for instance, may have been more likely to share positive experiences due to their connection with the group. The lack of representation of those who have chosen not to participate in support groups or who have had negative experiences with other professionals limits the generalization of the findings. This concentration in a single context could omit critical perspectives on the needs and challenges faced by other grieving parents. Therefore, future studies should seek to replicate and extend the present findings among more varied samples and support contexts.

## 5. Conclusions

In conclusion, the shared experiences of grieving parents highlight the need for an individualized and sensitive approach in the treatment of parental grief. Practices that prove beneficial not only alleviate suffering but also facilitate a healing process that allows parents to reframe death loss and rebuild their lives. However, it is crucial to avoid inadequate psychological practices that could exacerbate pain and hinder emotional adjustment. To improve support for grieving parents, effective communication, systematic assessment of needs, and ongoing follow-up are necessary to ensure that the support provided is perceived as respectful and valuable. Additionally, incorporating studies on policy interventions and cultural differences in bereavement can enhance the scope of support services, addressing the diverse needs of grieving parents across different contexts. Ultimately, the ability of professionals to integrate these individualized and culturally informed practices can transform the grieving experience, offering parents not only comfort but also a path toward hope and resilience amidst their pain.

## Figures and Tables

**Table 1:** Demographic data.

Participant	Sex	Age	Age of Son/Daughter at Death	Time Since Death	Cause of Death
1	Woman	60	20	6 years and 1 month	Allergy
2	Woman	50	15	2 years and 4 months	Cerebral hemorrhage
3	Woman	54	19	5 years	Car crash
4	Woman	57	19	3 years and 8 months	Suicide
5	Woman	56	18	2 ½ years	Accident
6	Woman	45	8	1 year	Inflatable Accident
7	Woman	63	17	13 years	Cancer
8	Woman	51	20	1 year and 3 months	Car crash
9	Woman	76	31	6 years	Lack of oxygen
10	Man	51	19	5 years	Car crash
11	Man	63	30	4 years and 9 months	Tetraplegia
12	Man	52	20	1 year and 2 months	Car crash
13	Woman	67	33	8 months	Bacterial meningitis
14	Woman	59	24	5 years and 10 months	Motorbike crash
15	Woman	59	26	1 year and 4 months	Car crash
16	Woman	72	44	1 year and 4 months	Suicide
17	Woman	50	21	2 years	Car crash
18	Woman	57	20	1 year and 7 months	Cancer
19	Man	59	20	1 year and 7 months	Cancer
20	Woman	72	34	9 years	Cancer
21	Woman	51	19	2 years	Cancer
22	Woman	63	23	6 years and 6 months	Car crash
23	Man	37	4	6 months	Drowning
24	Woman	59	26	1 year and 4 months	Car crash

## Data Availability

The data presented in this study are available on request from the corresponding author due to ethical, private and legal restrictions.
